# Size Analysis of Silver and Gold Nanoparticles Using Laser Ablation Single Particle ICP Mass Spectrometry: Evaluation of the Laser-Induced Disintegration of Nanoparticles

**DOI:** 10.5702/massspectrometry.A0116

**Published:** 2023-04-06

**Authors:** Shuji Yamashita, Takafumi Hirata

**Affiliations:** 1 Geochemical Research Centre, The University of Tokyo, 7–3–1 Hongo, Bunkyo-ku, Tokyo 113–0033, Japan

**Keywords:** spICP-MS, laser ablation, nanoparticle, size analysis, laser-induced disintegration

## Abstract

Single particle inductively coupled plasma mass spectrometry combined with the laser ablation technique (LA-spICP-MS) has been used for the determination of particle size and the spatial distribution of metal nanoparticles (MNPs) in various solid samples such as biological samples and semiconductor materials. In this study, we investigated the effect of the fluence of the laser being used on the disintegration of MNPs. Commercially available MNPs of silver and gold (Ag NPs and Au NPs), the sizes of which were determined by transmission electron microscopy (TEM), were analyzed with LA-spICP-MS. We evaluated the degree of disintegration of the original-sized particles, based on a comparison of the size distributions obtained by LA-spICP-MS and other analytical techniques. The disintegration of both the Ag NPs and Au NPs was induced by a laser ablation process when the laser fluence was higher than 1.0 J cm^−2^, whereas no disintegration was observed when the fluence was lower than 1.0 J cm^−2^. Moreover, the mean diameter and standard deviation of the determined diameters obtained by LA-spICP-MS were in good agreement with solution-based spICP-MS and TEM analysis within analytical uncertainty. The data obtained here demonstrates that LA-spICP-MS represents a promising potential analytical technique for accurately determining the size of individual MNPs and their spatial distribution in solid samples.

## 1. INTRODUCTION

Metal nanoparticles (MNPs) are widely used in various material, biological, and medical applications.^1–3)^ The rapid increase in the use of MNPs has resulted in these particles being released into the natural environment, leading to increased exposure on organisms. Physicochemical properties are related to particle size,^4)^ and thus, the characteristics and spatial distribution of MNPs in biological samples are important in terms of understanding the dynamics and toxicity of MNPs.^5,6)^

Single particle inductively coupled plasma mass spectrometry (spICP-MS) has recentrly emerged as an analytical technique for the detection of MNPs in solution samples, providing information on individual particle size and particle number concentrations.^7–9)^ During this analysis, MNPs enter the ICP, where the atoms contained in the MNPs are ionized, producing a burst of ions (one ion cloud per particle). The resulting ions enter the mass analyzer to be sorted by their mass-to-charge ratios, after which, the target ions enter the detector. Each particle is detected as a pulsed signal (particle event) with a particle event duration of between 200 to 1000 μs. Two types of information can be derived from these particle events: (i) individual particle size and (ii) particle number concentration. The signal intensity of each particle event reflects the number of detected ions derived from a single MNP, which is proportional to the volume, that is, the cubic value of the particle diameter. The frequency of detection of the particle events is proportional to the particle number concentration in the sample. The capability of spICP-MS has been demonstrated to be compatible with results obtained from transmission electron microscopy (TEM) and dynamic light scattering (DLS),^8)^ and thus, spICP-MS has now become a reliable technique for particle analysis.

For the *in-situ* characterization of MNPs in solid samples, spICP-MS combined with the laser ablation technique (LA-spICP-MS) is a promising analytical technique. LA-spICP-MS provides a variety of useful information, including individual particle size, the concentration of ionic form, and spatial distribution data of the MNPs and ionic form in solid samples. LA-spICP-MS was recently used to measure the spatial distribution of MNPs in biological samples.^10–12)^ Since the first application of LA-spICP-MS on particle analysis,^13)^ one of the most important considerations is the effect of the laser being used on the disintegration of MNPs to avoid the misreporting of particle size.

Among the various laser parameters, laser fluence (energy density: J cm^−2^) can particularly affect the disintegration of MNPs. It is reasonable to expect that the use of a low laser fluence would be needed to minimize the disintegration of MNPs. Previous studies revealed that a laser fluence lower than 1.0 J cm^−2^ would be appropriate for minimizing the disintegration of Au NPs during laser ablation.^14,15)^ In order to establish LA-spICP-MS as a practical analytical technique for particle analysis, a comparison of the particle sizes estimated from signal intensity by LA-spICP-MS and the sizes reported by other analytical techniques is needed. The focus of this study was on the effect of laser fluence on the disintegration of MNPs. The mean diameters and standard deviations of the determined diameters obtained from LA-spICP-MS were then compared to data obtained from solution-based spICP-MS as well as TEM analysis.

## 2. EXPERIMENTAL

### 2.1 Materials

Silver NPs (Ag NPs) suspensions with reported diameters of 20, 40, and 60 nm (NanoComposix, USA) and suspensions of gold NPs (Au NPs) with reported diameters of 20, 40, and 60 nm (NanoComposix, USA), stabilized in a citrate solution, were used in this study. The reported diameters determined by TEM analysis of these particles are listed in Table 1. A 10 μL portion of a particle suspension of each sample was dropped onto filter papers (qualitative filter paper No. 1, ADVANTEC, Japan), with sizes of 10 mm×10 mm. The filter papers were then allowed to dry at room temperature for 1 h, and the samples were then used for LA-spICP-MS analysis.

**Table 1:** Table 1. Reported diameters of commercially available nanoparticles.

Sample	Diameter (nm)
20 nm Ag NPs	20.8±3
40 nm Ag NPs	41±5
60 nm Ag NPs	59±6
20 nm Au NPs	19.3±2.1
40 nm Au NPs	39±4
60 nm Au NPs	60±6

### 2.2 Instrumentation

The ICP-MS instrument used in this study was a single collector-magnetic sector-based ICP-MS (AttoM, Nu Instruments, UK). The low mass resolution mode (*m*/Δ*m*=300) was adopted so as to obtain maximum ion transmission. For the solution-based spICP-MS, a desolvation sample introduction system (Aridus II, Teledyne Cetac Technologies, USA) was used to simulate dry plasma conditions, which is a similar plasma condition to that when the laser ablation technique is applied.

For the laser ablation system, a Nd:YAG laser (MOPA 266–200 mW, CryLas, Germany) was employed. The laser fluence applied in this study was 0.2 to 3.0 J cm^−2^, which was the minimum and maximum fluences in the laser ablation system. The size of the laser beam cannot be changed in the case of the laser ablation system used this study, and thus, default parameter for the laser beam size of 5 μm was applied. The prepared filter paper samples (prepared by dropping suspensions of Ag NPs and Au NPs on them) were subjected to laser ablation line scanning. The laser-induced aerosols were transported to the ICP with a mixed gas, with helium as the carrier gas and argon gas as the makeup gas. Argon gas was added after the ablation cell to stabilize the plasma.

For the ICP-MS system used in this study, ion detection is usually conducted in the pulse counting mode with electron multiplier, and at ion count rates higher than 3×10^6^ cps, ion detection switches to the analog mode with a Faraday detector. However, the response of the Faraday detector requires about 100 ms, which is longer than a particle event duration.^16)^ This causes the inaccurate acquisition of particle events due to the Faraday detector being unable to keep up with the rapid changes in the signal intensities produced by the particles.^16)^ In order to detect ions using the pulse counting mode even from high count rates, a platinum grid (attenuator) is installed to physically attenuate the ion beam of high count rates prior to its entrance into the electron multiplier. The attenuator system has the advantage of using pulse counting mode even from high count rates (*e.g.*, 3×10^7^ cps) derived from particles while maintaining the speed of data acquisition.^17)^ It should be noted that count rates higher than 1×10^6^ cps will be attenuated down to the 1/500 level by passing through the attenuator, while count rates lower than 1×10^6^ cps travel to the detector *via* another pathway without the attenuator. The attenuator system runs automatically. Hence, the upper limit of detection in the pulse counting mode can be extended from 3×10^6^ to 1.5×10^9^ cps, without sacrificing sensitivity of low ion count rates.^17)^ Details of the instrumentation and operational settings are listed in Table 2.

**Table 2:** Table 2. Instrumentation and operational settings.

ICP-MS	
Instrument	Magnetic sector-based ICP-MS (AttoM, Nu Instruments, UK)
ICP forward power	1300 W
Argon gas flow rate	
Coolant gas	13.0 L min^−1^
Auxiliary gas	1.0 L min^−1^
Monitored isotopes	^107^Ag and ^197^Au
Dwell time	50 μs
Detector	ETP full size multiplier
Detection system	Pulse counting mode with attenuator system
Desolvation sample introduction system	
Instrument	Aridus II (Teledyne Cetac Technologies, USA)
Sample uptake rate	0.2 mL min^−1^
Spray chamber temperature	110°C
Membrane oven temperature	160°C
Sweep gas	Ar (4.8 L min^−1^)
Laser ablation system	
Instrument	Nd:YAG laser (MOPA 266–200 mW, CryLas, Germany)
Wavelength	266 nm (FHG)
Pulse width	1 ns
Fluence	0.2 to 3.0 J cm^−2^
Repetition rate	10 Hz
Carrier gas	He (0.60 L min^−1^)
Makeup gas	Ar (1.0 L min^−1^) mixed after the ablation cell using T-piece connector
Scanning speed	100 μm s^−1^
Laser beam size	5 μm

### 2.3 Data collection

Conventional ICP-MS systems consist of two data acquisition parameters: a dwell time (reading time for the signal) of more than 1 ms and a settling time (overhead and processing time). After the measurement period (dwell time), approximately 1 ms is spent for the settling time before the next measurement is performed. If ions reach the detector during the settling time, they will not be detected, leading to the inaccurate counting of ions. Since particles are detected as pulsed signals with a particle event duration between 200 to 1000 μs, ion clouds derived from a single particle will either be missed entirely or detected as partial events.^18)^ Thus, the elimination of settling time is important for single particle analysis using ICP-MS.

Another important requirement for the measurement of particles with spICP-MS is a fast data acquisition system (*i.e*., a system capable of measurement with a dwell time shorter than a particle event duration). The choice of dwell time will depend on (i) the ability to acquire pulsed signals from single particles with a high peak height to distinguish them from background signals and (ii) preventing the probability of multiple particles being measured within a single dwell time (coincidence) and overlapped particle detection. The problem of adopting very short dwell times (*e.g.*, 10 μs, which is the shortest dwell time achievable by ICP-MS instruments) is that the detection of particles is not appropriate because the detection of ions per dwell time will be low.^19)^ Whereas, the probability of coincidence and overlapped particle detection increases when the dwell time is longer than 100 μs.^18–20)^ From previous studies on the effect of dwell time, continuous and data acquisition with dwell times of 30 to 100 μs is ideal.^18–20)^ Based on these factors, data acquisition was conducted with a dwell time of 50 μs in this study.

### 2.4 Data processing

In order to obtain reliable particle sizes using spICP-MS, pulsed signals derived from single particles should be distinguished from background signals. The detection limit was set as μ +*5*σ, where the μ and σ values correspond to the mean signal intensity and the standard deviation of the baseline of the time scan, respectively.^21)^

Each particle size is calculated based on the signal intensity of each particle event. Size calibration based on a calibration curve constructed with multiple particle size standards is ideal.^22)^ However, since it is difficult to obtain multiple particle size standards with a defined particle diameter and monodispersed distribution for most elements, demonstrating size calibration using only one particle size standard is important. In previous studies, including our group, size calibration using only one particle size standard was evaluated, and accurate size information was obtained.^23,24)^ We therefore concluded that the size calibration method using one particle size standard is an effective approach for obtaining reliable size data.

Also, in this study, size calibration using only the one particle size standard was re-evaluated. An in-house software (NanoQuant) was used to calculate the signal intensity of individual particle events.^24,25)^ The calibration factor *f* (nm^3^/counts per particle event) was defined by relating the particle size of the particle size standard and the signal intensity of the particle event obtained, as shown in Eq. (1) 
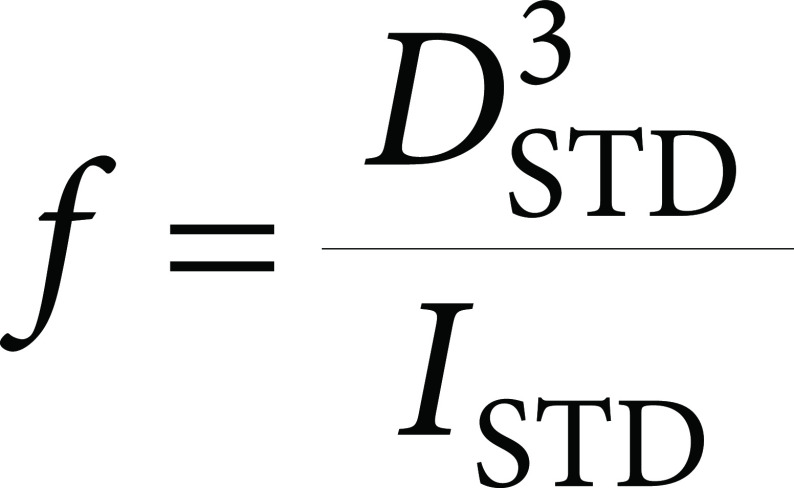
(1) where *D*_STD_ is the nominal particle size (in diameter) and *I*_STD_ represents the signal intensity of a single particle event corresponding to the nominal particle size. The *D*_STD_ value was the mean diameter of the TEM data provided by the manufacturer. The *I*_STD_ value was determined as the most frequent signal intensity of the lognormal distributions obtained during analyzing the particle size standard.^24)^

## 3. RESULTS AND DISCUSSION

### 3.1 Size analysis using solution-based spICP-MS and LA-spICP-MS

In order to study the disintegration of MNPs induced by the laser ablation process, size analysis using LA-spICP-MS was investigated by comparing its size distribution with values obtained using solution-based spICP-MS. 60 nm MNPs were first measured to determine the calibration factor *f*. Based on the signal intensity *I*_STD_ and the reported diameter *D*_STD_, the calibration factors for solution-based spICP-MS were 123.8 nm^3^/counts per particle event and 41.0 nm^3^/counts per particle event for Ag NPs and Au NPs, respectively; while for LA-spICP-MS, these values were 167.3 nm^3^/counts per particle event and 73.1 nm^3^/counts per particle event for Ag NPs and Au NPs, respectively. The particle sizes of 40 nm MNPs were calibrated by using the *f* values stated above. The fluence applied in this experiment was 0.2 J cm^−2^.

**Figure 1:**
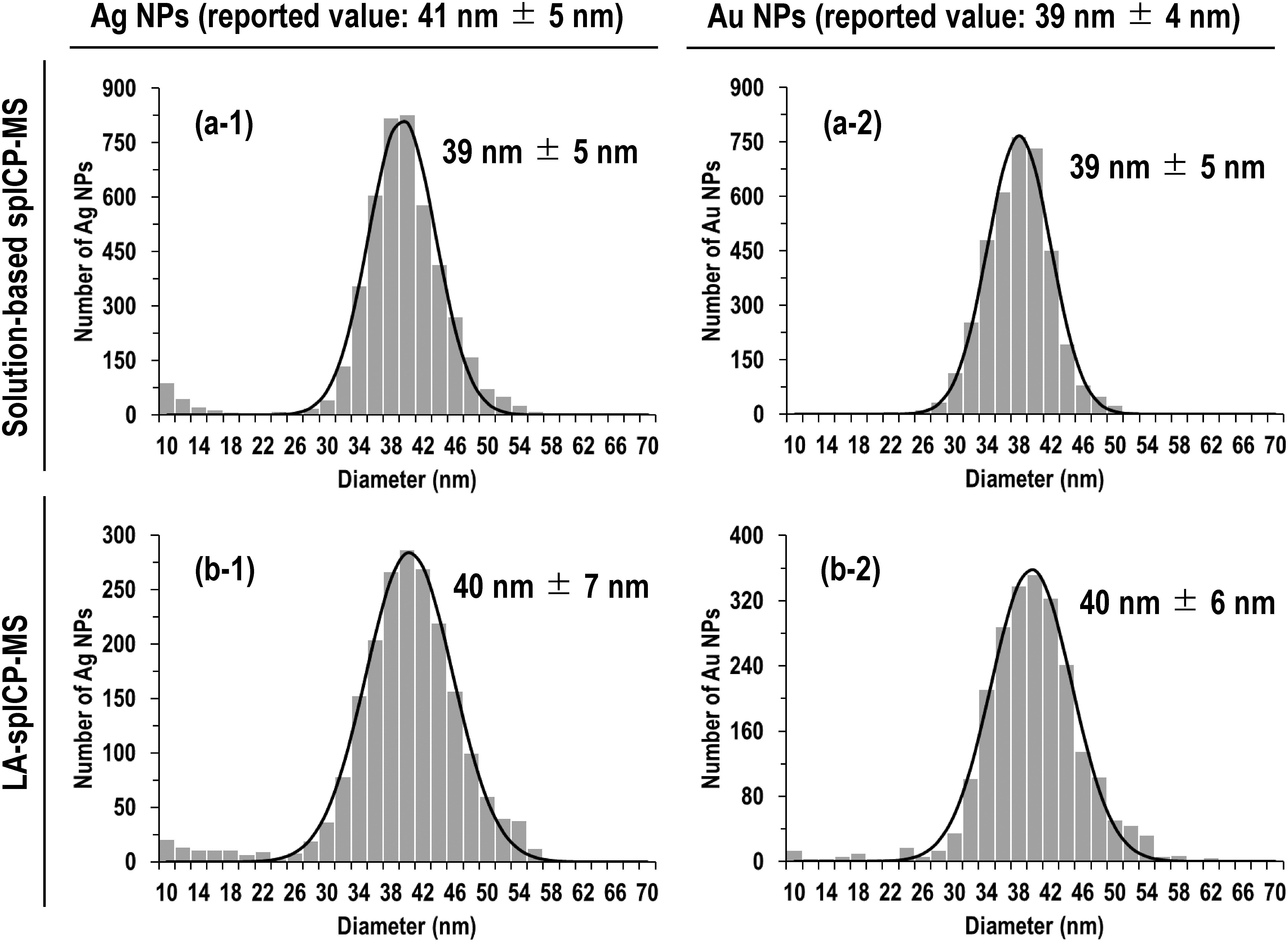
Fig. 1. Size distributions for the measured (a-1) 40 nm Ag NPs (*N*=4611 particle events) and (a-2) 40 nm Au NPs (*N*=3846 particle events) using solution-based spICP-MS. Size distributions for the measured (b-1) 40 nm Ag NPs (*N*=2038 particle events) and (b-2) 40 nm Au NPs (*N*=2371 particle events) using LA-spICP-MS with a laser fluence of 0.2 J cm^−2^. These size distributions were determined by calibration with 60 nm Ag NPs and Au NPs as particle size standards.

Figure 1 illustrates the size distributions of 40 nm Ag NPs obtained by solution-based spICP-MS (Fig. 1a-1) and LA-spICP-MS (Fig. 1b-1). The size distributions were fitted with a Gaussian distribution in the range from 20 to 60 nm. The size distribution obtained by the solution-based spICP-MS displayed a monodisperse distribution with the mean diameter and standard deviation of the determined diameters of 39±5 nm. The size distribution estimated using LA-spICP-MS was also monodispersed, with the mean diameter and standard deviation of the determined diameters of 40±7 nm. There was no significant difference between the size distributions obtained by the solution-based spICP-MS and LA-spICP-MS. More importantly, for the LA-spICP-MS, there was no significant increase in the number of Ag NPs with sizes less than 30 nm. The ratio of the number of 10 to 30 nm Ag NPs to the total number of Ag NPs were 5.9% for solution-based spICP-MS and 7.4% for LA-spICP-MS, respectively, suggesting that the laser-induced disintegration at a fluence of 0.2 J cm^−2^ made only a small contribution.

**Figure 2:**
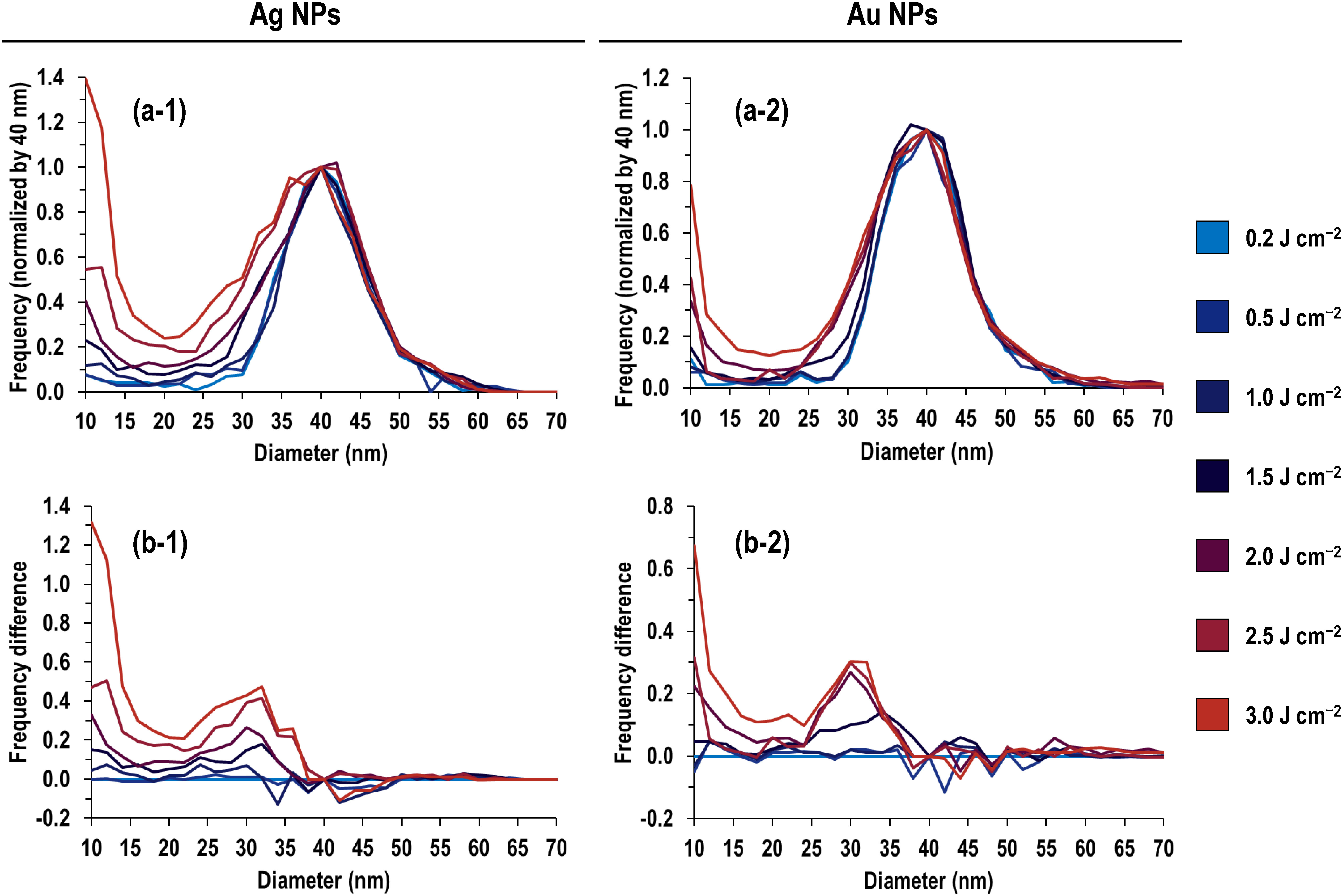
Fig. 2. Size distributions of (a-1) 40 nm Ag NPs and (a-2) 40 nm Au NPs as a function of laser fluence after data processing. Size distributions for each fluence after subtraction of 0.2 J cm^−2^ for (b-1) 40 nm Ag NPs and (b-2) 40 nm Au NPs.

Figures 1a-2 and 1b-2 show the size distributions of 40 nm Au NPs obtained using solution-based spICP-MS and LA-spICP-MS, respectively. The size distributions were fitted with a Gaussian distribution in range from 20 to 60 nm. For the Au NPs, both techniques showed monodispersed distributions. The mean diameter and standard deviation of the determined diameters were 39±5 nm for the solution-based spICP-MS, and 40±6 nm for the LA-spICP-MS. Both techniques were in agreement within the analytical uncertainties for the mean diameter and standard deviation of the determined diameters. Similar to Ag NPs, no significant increase in the Au NPs of smaller sizes (<30 nm) was found when LA-spICP-MS was used. The ratio of the number of 10 to 30 nm Au NPs to the total number of Au NPs was 4.8% for solution-based spICP-MS and 5.0% for LA-spICP-MS, respectively. From these data, regardless of the chemical compositions, the size distributions obtained by LA-spICP-MS were similar to those obtained by solution-based spICP-MS when a laser fluence of 0.2 J cm^−2^ was applied.

### 3.2 Effect of fluence on size distribution

The disintegration of MNPs during the laser ablation process will lead to systematic errors for size analysis, and thus, optimal laser fluence needed to minimize the disintegration of MNPs was investigated. As mentioned above, 60 nm Ag NPs and 60 nm Au NPs were used as particle size standards for the calibration of their respective elements. The calibration factors were 167.3 and 73.1 nm^3^/counts per particle event for the Ag NPs and the Au NPs, respectively. It should also be noted that the *f* value was obtained with a fluence of 0.2 J cm^−2^ (lowest adjustable fluence with the laser ablation system) under the assumption that the contribution of laser-induced disintegration was negligible. The resulting *f* was also applied for the size analysis conducted by other fluences.

Figures 2a-1 and 2a-2 illustrate the size distributions for 40 nm Ag NPs and 40 nm Au NPs obtained with various laser fluences ranging from 0.2 to 3.0 J cm^−2^. The size distributions were defined based on a total of about 1000 particle events, and the frequency was normalized with that of 40 nm particles to compare between the distributions obtained by the different fluences. For laser fluences ranging from 0.2 to 1.0 J cm^−2^, the resulting mean diameters did not vary significantly, suggesting that laser-induced disintegration was a minor contributor. In contrast, the frequency between the 10 nm particles (*i.e*., disintegrated MNPs) and the 40 nm particles (*i.e*., original-sized particles) in size distribution becomes higher with increasing laser fluence.

Figures 2b-1 and 2b-2 illustrate the frequency difference after subtracting the size distribution of 0.2 J cm^−2^. Negative values in the frequency difference within 35 to 50 nm were found; while the frequency difference was positive for particles smaller than 35 nm (*e.g.*, 3.0 J cm^−2^). These results suggest that the original MNPs with sizes of 35 to 50 nm had disintegrated by laser ablation and smaller-sized particles derived from the disintegrated original-sized particles were produced. Based on these results, when a fluence higher than 1.0 J cm^−2^ was adopted, the number of original-sized particles would decrease and smaller-sized particles would increase.

In Figs. 2b-1 and 2b-2, there are two peaks, one at 10 nm, and the other at around 30 nm. The reason for why two peaks were generated can be attributed to the different laser energy absorbed by the analyzed MNPs. The laser ablation system used in this study exhibited a Gaussian beam profile (*i.e.*, uneven laser energy distribution with a higher energy distribution at the center and a lower energy distribution along the rim). Hence, it would be expected that the laser energy would not be applied uniformly to the irradiated area. Since MNPs are distributed randomly in the prepared filter paper samples, there will be some particles that absorb a higher laser energy while others would absorb a lower laser energy. Based on this reasoning, the peak at 10 nm may be due to original-sized particles being more extensively disintegrated when a higher laser energy is absorbed; while the peak at 30 nm may be due to the partial disintegration of original-sized particles when a lower laser energy is absorbed.

Moreover, in Figs. 2b-1 and 2b-2, the frequency of particles around 10 nm are higher than 30 nm. Assuming that the 40 nm particles disintegrate uniformly, the number of disintegrated particles will be the ratio of the cubic value of 40 nm to that of the disintegrated particles. For example, a single 40 nm particle will disintegrate into 64 particles of 10 nm particles ((40/10)^3^=64), 8 particles of 20 nm particles ((40/20)^3^=8), and about 2 particles of 30 nm particles ((40/30)^3^=2.3), respectively. In other words, the number of disintegrated particles generated increases with decreasing particle diameter. The peak at around 30 nm in both Figs. 2b-1 and 2b-2 indicates that if a single 40 nm particle is disintegrated into two equal particles, two 31.7 nm particles would be generated because volume is proportional to the number of atoms. This explains why the frequency at around 10 nm is higher than that at 30 nm.

Nevertheless, it should be noted that larger-sized particles (>50 nm) were observed regardless of the fluence (Figs. 2b-1 and 2b-2). As an example, if two 40 nm particles aggregate, a single 50 nm particle would be generated ((50/40)^3^=1.95). 40 nm particles were the dominant particles used in this experiment, and thus, the possibility of two 40 nm particles undergoing aggregation was high. Since the number of particles larger than 50 nm in solution-based spICP-MS were not as high as those for LA-spICP-MS (Fig. 1), aggregation/agglomeration of the Ag NPs and Au NPs may have occurred on the filter paper during the drying process, due to the similar frequency of particles larger than 50 nm, irrespective of fluence.

**Figure 3:**
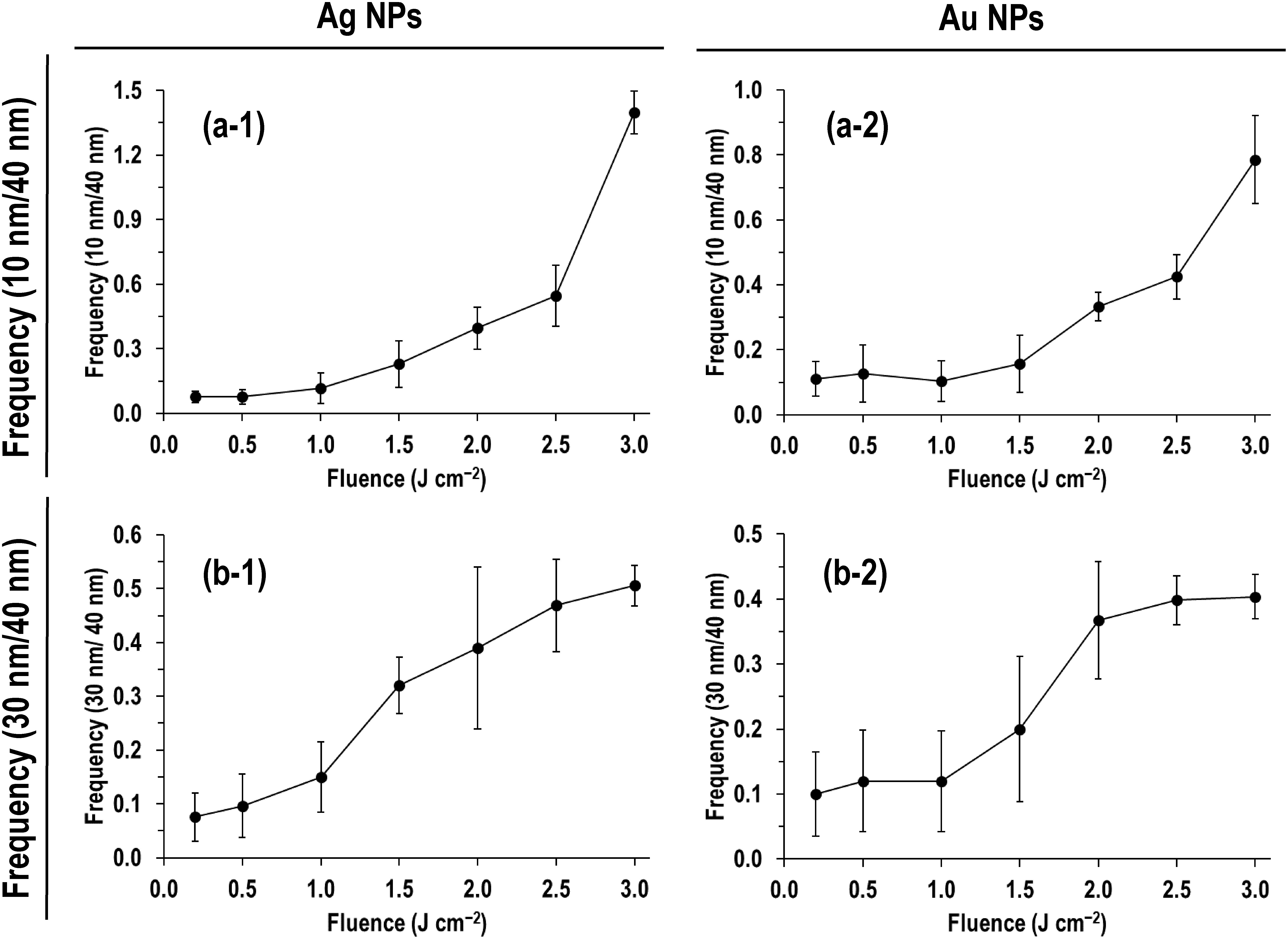
Fig. 3. Ratio of 10 nm particles to 40 nm particles plotted against the laser fluence for (a-1) Ag NPs and (a-2) Au NPs. Ratio of 30 nm particles to 40 nm particles plotted against the laser fluence for (b-1) Ag NPs and (b-2) Au NPs.

Figures 3a-1 and 3a-2 show the ratio of 10 nm particles to 40 nm particles to evaluate the disintegration of Ag NPs and Au NPs, respectively. Figures 3b-1 and 3b-2, the ratio of 30 nm particles to 40 nm particles of Ag NPs and Au NPs, respectively. All figures in Fig. 3 show that the increase in the ratio of disintegrated particles-to-original-sized particles is insignificant from 0.2 to 1.0 J cm^−2^ but increase significantly after 1.0 J cm^−2^. From these data, regardless of the chemical compositions, a fluence lower than 1.0 J cm^−2^ is required to minimize the contribution of the laser-induced disintegration of MNPs.

### 3.3 Correlation between signal intensity of particle event and diameter

An effective approach for evaluating data quality in a size analysis using spICP-MS is to investigate the correlation between the signal intensity of the particle event and the diameter.^24)^ Since the MNPs used in the present study are spherical, the volume is equal to 4/3π(*d*/2)^3^ (*d*: diameter). Here, the number of atoms contained in a single particle is proportional to volume, that is, the cubic value of the particle diameter. This can be confirmed from the regression line with a slope of three on the log-log plot of particle diameter against signal intensity.^24)^

**Figure 4:**
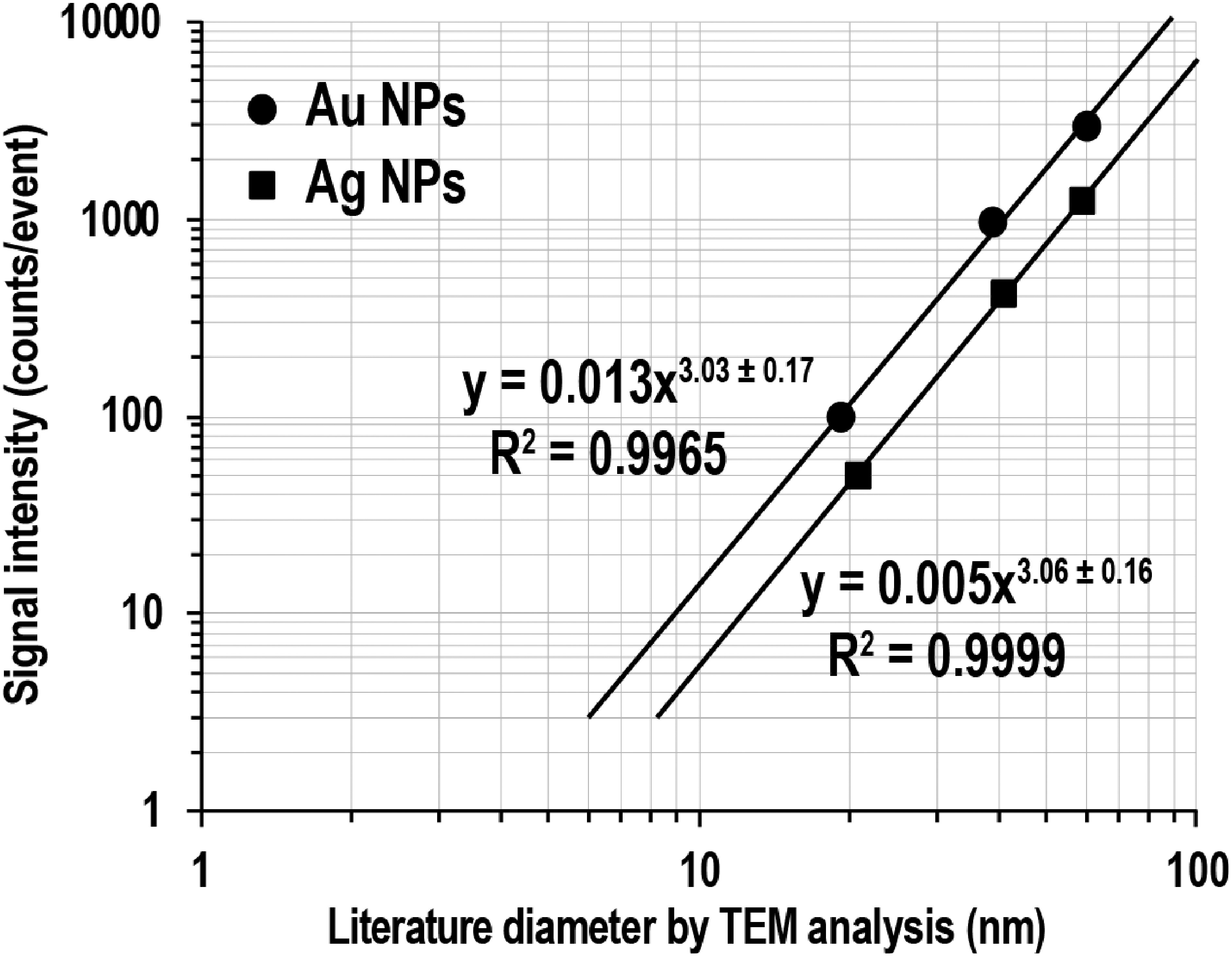
Fig. 4. Signal intensities (counts per particle event) plotted against the diameters of Ag NPs and Au NPs determined by the TEM analysis. The signal intensities are the mode values calculated from the lognormal-fitted signal intensity distributions.

For this validation, laser ablation with a fluence of 0.2 J cm^−2^ was performed on various sizes of Ag NPs and Au NPs, and the signal intensity of individual particle events were measured. Figure 4 shows signal intensity of Ag NPs (closed square) and Au NPs (closed circle) of various diameters plotted against the reported diameters determined by TEM analysis. The signal intensity of each data point was determined by the mode value calculated from the lognormal-fitted signal intensity distributions,^24)^ and were used to construct a regression line. The data points defined a regression line with a slope of 3.06±0.16 (standard error) for Ag NPs and 3.03±0.17 (standard error) for Au NPs, showing that the measured signal intensity was correlated with the number of atoms in single particles. These correlations demonstrate the validity of size distribution measurements for MNPs obtained by LA-spICP-MS. We therefore conclude that, regardless of size and chemical composition, the ionization and transmission efficiencies of ions passing through the mass spectrometer remain relatively constant for each particle samples.

## 4. CONCLUSION

The focus of this study was on the effect of laser fluence on the disintegration of Ag NPs and Au NPs. The size distributions of Ag NPs and Au NPs that were determined by LA-spICP-MS were comparable to the values determined by solution-based spICP-MS and TEM analysis. In addition, the frequency of particles smaller than 35 nm increased when fluences were higher than 1.0 J cm^−2^. From these results, we conclude that regardless of chemical compositions, a fluence lower than 1.0 J cm^−2^ is required to minimize the contribution of the laser-induced disintegration of MNPs. Moreover, the data points for both Ag NPs (20 nm, 40 nm, and 60 nm) and Au NPs (20 nm, 40 nm, and 60 nm) were defined a regression line with a slope of three on the log-log plot of particle diameter against signal intensity of the particle event. This result demonstrates the validity of size distributions for MNPs obtained by LA-spICP-MS.

In recent years, biochemists have increasingly become interested in the behavior of MNPs in biological samples: “how MNPs interact with organs and cells” or “what MNP size results in toxicity.”^6)^ In order to develop better understanding of the biological effects of MNPs, sensitive analytical techniques for the imaging and size analysis of MNPs are desired. For the imaging and accurate size analysis of MNPs in biological samples using LA-spICP-MS, both sufficient ablation of biological samples and the suppressing the disintegration of MNPs within the samples are required. Biological samples are sufficiently ablated with a fluence at around 1.0 J cm^−2^.^12,26)^ Taking our results into consideration, both a sufficient ablation of biological samples and minimizing the disintegration of MNPs can be simultaneously achieved when a fluence lower than 1.0 J cm^−2^ is applied. We therefore conclude that LA-spICP-MS represents a useful technique for investigating the behavior of MNPs within biological samples.

## Conflicts of Interest

There are no conflicts to declare.

## Notes

Mass Spectrom (Tokyo) 2023; 12(1): A0116
